# Exploring the Role of Healthy Green Spaces, Psychological Resilience, Attitude, Brand Attachment, and Price Reasonableness in Increasing Hotel Guest Retention

**DOI:** 10.3390/ijerph17010133

**Published:** 2019-12-23

**Authors:** Jongsik Yu

**Affiliations:** College of Hospitality and Tourism Management, Sejong University, 98 Gunja-Dong, Gwangjin-Gu, Seoul 143-747, Korea; andyjs.yu@gmail.com; Tel.: +82-2-3408-4462

**Keywords:** green spaces, psychological resilience, hotel guests, brand-self connection, brand prominence, retention, hotel price

## Abstract

The present research was an empirical endeavor to explore the effect of green spaces on the traveler retention process and to establish a theory connecting such green spaces, psychological resilience, attitude, brand attachment, and retention in the hotel industry. A quantitative approach was employed to achieve study objectives. Our findings from the structural analysis indicated that green spaces as nature-based solution significantly influence psychological resilience. In addition, such relationship contributes to increasing positive attitude, strengthening brand-self connection and brand prominence, and building traveler retention. A salient role of attitude in determining retention was found. A further analysis (metric invariance) revealed that the linkage from green spaces to psychological resilience was moderated by hotel price reasonableness, and the association became stronger when guests feel that hotel price is reasonable. Overall, this research successfully verified the importance of a hotel’s green spaces and its role in guest psychological and affective responses and behaviors.

## 1. Introduction

The concept of nature-based solutions is a growing global phenomenon that helps societies effectively address diverse social and environmental challenges in a sustainable way [1]. This concept uses the potential power of nature as a means of providing solutions for such challenges [2,3]. Nature-based solutions are often considered to be an important contributor to sustainable development in many regions globally [1,4]. Due to its sustainable characteristics and pursuit of societal and environmental benefits, nature-based solutions, in recent years, have received increasing attention in various contexts [4,5,6]. A society that employs nature-based solutions uses nature (or “greening”) to address various challenges, with favorable outcomes for the society [1].

Within a building that was designed in a sustainable or “green” way, a good example of nature-based solution efforts is green spaces that positively affect the responses and actions of the building’s occupants [7]. Similarly, an example of a hotel’s nature-based solution endeavors is creating or increasing green spaces within the building and outside [6]. A company’s green spaces, along with eco-friendly physical environments, often play a critical role in positively affecting the mental health and perceptions of well-being to induce positive behaviors among a company’s personnel and its customers [2,8]. Likewise, to increase feelings of health and psychological well-being in internal and external customers, it is imperative that companies should create green spaces within and outside a building which are easily accessible and readily available.

Operating a hotel in an environmentally responsible way is regarded as a major issue in the global hotel industry [9,10,11]. However, few existing studies have reported the possible outcomes of a hotel’s green spaces as a nature-based solution. Moreover, while the significance of psychological resilience, attitude, brand-self connection, and brand prominence has often been emphasized in environmental behavior and marketing literature [12,13,14,15,16,17], minimal research has addressed the potential relationships among these variables to provide a clear understanding of the possible impact of these associations on the hotel traveler retention process. Moreover, the criticality of the concept of “price reasonableness” and its effect on customers’ post-purchase decision formation has been emphasized in recent literature [18,19,20]. Despite its importance, hotel guests’ perception of price reasonableness and its influence have rarely been investigated to explain the formation of guests’ psychological resilience. Furthermore, while researchers of consumer behavior and tourism often consider the moderating nature of price reasonableness [18,21], little is known of how it moderates or of the effect of a hotel’s green spaces. 

This study aims to fill these gaps in the existing literature. It also aims to uncover the role of green spaces as nature-based solutions for inducing psychological resilience and to discover the possible influence on the formation of traveler retention in the hotel industry. Moreover, this study was designed to: (1) explore the role of attitude, brand-self connection, and brand prominence as direct and indirect determinants of traveler retention, (2) identify the comparative importance among study variables in generating retention, and (3) uncover the moderating effect of hotel price reasonableness. In summary, this research is an empirical endeavor to build a sturdy conceptual framework that includes green spaces as nature-based solutions, psychological resilience, attitude, brand-self connection, brand prominence, and price reasonableness. These concepts have never been applied together in an attempt to comprehend traveler retention formation in the hotel industry. 

## 2. Conceptual Framework

### 2.1. Green Spaces as Nature-Based Solution Efforts

While the concept of nature-based solutions is an emerging topic in environmental research, it is a relatively a new term in the business sector. Nature-based solutions refer to a sustainable approach using the natural environment as a means of providing effective solutions to a number of challenges in society and the environment while simultaneously improving societal and environmental outcomes [1,6]. Nature-based solutions can enhance air quality, decrease noise pollution, improve water quality, reduce pollution, and decrease possible natural disasters, all of which are beneficial for the environment [3,4]. Moreover, human lives can be changed in healthy and positive ways through green spaces and natural-based solution efforts [2,4]. Specifically, there are positive effects achieved, such as reducing stress, increasing physical energy, reducing heat inside buildings, improving air quality, and preventing mental and cardiovascular diseases with the nature-based solution strategy, including the formation of green spaces [4].

One of the core constituents of nature-based solutions’ effects are green spaces [4,7]. Green spaces include specially designed places containing gardens, plants, and natural features inside and outside a building. These places are normally designed for rest, leisure, and physical activities for the building’s occupants (e.g., customers or employees). Diverse green spaces (e.g., green rest areas, gardens, natural surroundings, swimming pools, or green lobbies) are often available at hotels [2,6,7]. These types of spaces, and the natural environment in general, undoubtedly influence humans, generating outcomes of psychological resilience and well-being, as well as providing physical health benefits [4,22,23]. Such physical surroundings also elicit either approach responses or avoidance responses among the occupants of a building (e.g., customers, visitors, or employees) [24,25,26]. This is particularly relevant for customers, since approach responses are comprised of different positive behaviors that are directed at a specific brand or product (e.g., retention, loyalty, recommendation, citizenship behaviors, and willingness to pay), and firms actively attempt to fortify customers’ approach responses [24,26].

### 2.2. Psychological Resilience

Mental health is undeniably a fast-growing and important issue globally due to the sharp increase in the number of people struggling with mental health problems in recent times (e.g., anxiety, stress, worrying, depression, relationship problems, self-distrust, emotional disorders, and diffidence) [7,22,27]. Self-rated mental health refers to an individuals’ cognitive assessment of their own current mental health condition [28]. Moreover, psychological resilience is an important aspect of individual mental health perception [14]. Psychological resilience can be described as an individual’s self-evaluation regarding their ability to handle a mental crisis (e.g., mental stress or anxiety) or to come back to pre-crisis status (e.g., feel refreshed or relieved). Individuals’ psychological resilience protects them from the potential harmful impacts of stressors [28] and individuals with psychological resilience are likely to maintain a healthy balance between diverse life activities (e.g., work, family, and leisure) and mental well-being [14,15]. The psychological resilience and mental health of both customers and employees are also critical issues in global business. Existing research has revealed that individuals’ exposure to green spaces and natural atmospheres leads to increased psychological resilience and improved mental health [7,15]. Specifically, a green environment has a positive impact on people’s mental and physical health, as well as contributes greatly to enhancing an area’s healing ability, with which people can recover from psychological burdens, such as stress and depression [29,30]. In addition, therapies using the natural environment are improving people’s lives by promoting emotional health, enhancing welfare, achieving emotional recovery, and providing new business opportunities [31]. In this regard, green and natural environments have significant healing effects.

**Hypothesis** **1** **(H1).** *Green spaces have a significant influence on psychological resilience*.

### 2.3. Attitude

Attitude is a well-established concept that has been broadly researched in existing literature because of the possible benefits it brings to a business [12,32,33]. Increased brand/product attachment, loyalty, retention rate, and revenue—which are all directly related to a company’s business success—are often regarded as outcomes of customers’ positive attitude toward the company and its products or services [2,34,35]. Therefore, many business operators endeavor to increase customers’ favorable attitude toward their companies in diverse ways [12,13,16]. Attitude refers to the degree to which a customer has a positive or negative assessment when using a specific product [33,36,37]. 

Like in many sectors, attitude is a major component of the customer retention process in the hotel industry [13,32,33,34], and its critical role in clarifying hotel guests’ post-purchase behaviors and retention formation has been greatly emphasized [12,32,33]. These studies all agree that the role of attitude becomes more apparent in explaining guest behaviors concerning environmentally responsible hotel products. Customers’ attitudes toward a company is undoubtedly influenced by individuals’ outcome beliefs (e.g., mental health and psychological well-being/resilience) based on eco-friendly hotel companies’ greening efforts and environmentally responsible management. In particular, hotels’ nature-friendly physical environment can have a close relation with customers’ attitude and behavior [38]. Bitner [24] argued that the physical environment contains various characteristics influencing a person’s senses, and therefore, customers show cognitive, emotional, and physiological responses; through such responses, the physical environment directly affects customers’ attitude and behavior. As such, a nature-friendly physical environment improves emotional health and the level of comfort of indoor residents. Therefore, increasing attention has been paid to the quality and importance of the environment [7]. Such an attitude is likely to boost guests’ connection and commitment to that particular hotel brand [2,32,34] and its products, thereby increasing retention rate [12,13,33].

**Hypothesis** **2** **(H2).** *Psychological resilience has a significant influence on attitude*.

**Hypothesis** **3** **(H3).** *Attitude has a significant influence on brand-self connection*.

**Hypothesis** **4** **(H4).** *Attitude has a significant influence on brand prominence*.

**Hypothesis** **5** **(H5).** *Attitude has a significant influence on traveler retention*.

### 2.4. Brand-Self Connection and Brand Prominence

Attachment is frequently depicted as a core constituent of existing post-purchase behavior frameworks [17,25,35,39]. According to the attachment theory [40], brand attachment indicates the strength of the bond or connection between an individual (a customer) and the brand. Brand attachment generally includes brand-self connection and brand prominence as its key dimensions [16,40]. From a marketing perspective, Park et al. [16] described brand-self connection as cognitive and affective bonding. A customer builds a feeling of oneness with the brand, developing cognitive and affective connections that link the brand with the self [17]. Mikulincer [41] described brand prominence as positive memories or feelings toward the attachment object (brand) perceived as the top of mind. Such positive memories/feelings are more salient for individuals who are strongly attached to it than for those who are weakly attached to it [41]. These components often serve as core indicators of brand attachment [16,17].

Recently, researchers have been focusing on the critical role of brand-self connection and brand prominence in explaining customers’ consumption behaviors and retention processes [16,25]. Existing empirical evidence on consumer behavior, marketing, and hospitality has shown that customer attachment to a particular brand, product, or place significantly enhances the likelihood of customers repurchasing or revisiting and a willingness to be loyal to the brand, product, or place [16,17,25,35,41,42]. Concerning customers’ choices of hospitality or tourism products and services, the concept of attachment (brand-self connection and brand prominence) and its role in triggering customer retention becomes even more critical, as such products and services have little tangible cues to rely on [25]. Overall, the findings of these existing studies were in line with that of Mikulincer [41] and Mikulincer and Shaver’s [40] earlier assertion regarding the criticality of brand attachment in customer post-purchase behaviors.

**Hypothesis** **6** **(H6).** *Brand-self connection has a significant influence on traveler retention*.

**Hypothesis** **7** **(H7).** *Brand prominence has a significant influence on traveler retention*.

### 2.5. Hotel Price Reasonableness

In consumer behavior and tourism, price is a prevailing tool utilized for the maximization of a firm’s profits and its customer retention rate [19,21,43]. From customers’ perspectives, the price of a product or service is best described as their sacrifice to obtain/use the product/service [43]. Price is also considered as a critical indicator of product/service quality [43]. According to Oh [44] and Petrick [45], price reasonableness refers to customers’ perceptions that are based on the comparison between the actual product price and reference prices (e.g., commonly paid price from their preceding pricing encounter, price of similar offerings by competitors, or market prices). When customers believe that the price is lower than the reference prices, their perception of the price reasonableness increases [44,46]. However, when the opposite is presented, customers are likely to perceive the price as not reasonable [46]. In the global hospitality industry, due to ever-increasing competition, guests and their choices are largely influenced by their perception of hotel price reasonableness [19,21,47].

Previous studies have emphasized the significance of price reasonableness in explaining customer behavior [19,20,46,47]. These studies indicate that customers’ post-purchase decision formation concerning hospitality/tourism products is largely dependent on the cognitive aspect of price perception. Undoubtedly, hotel customers are becoming increasingly aware of whether a hotel offers reasonable rates and if staying at the hotel is worth its price [19]. Green spaces and natural-based solution efforts have a significant positive impact on human welfare and psychological resilience, as well as considerable potential for providing new business to hotels and solving a variety of social problems [1,6,22]. This impact can be maximized if customers perceive a high price reasonableness, as reasonable prices often fortifies customers’ positive experience with a product or service and its effect on subsequent factors [19,20,21,46]. While research investigating the moderating role of price reasonableness in the hotel sector is not abundant, based on the evidence of the studies discussed above, the following hypothesis is proposed:

**Hypothesis** **8** **(H8).** *Hotel price reasonableness significantly moderates the relationship between green spaces and psychological resilience*.

### 2.6. Proposed Conceptual Framework

**Figure 1:**
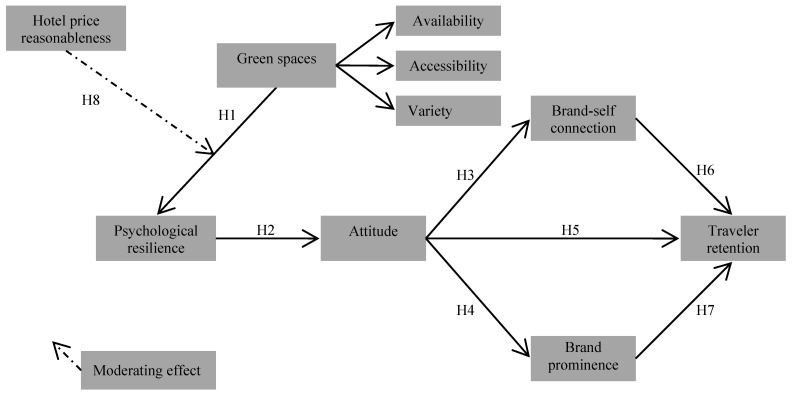
Proposed conceptual model and research hypotheses.

The proposed model and the research hypotheses are presented in Figure 1. The theoretical framework contains seven hypotheses linking the research constructs (Hypotheses 1–7). It also includes one hypothesis regarding the moderating effect of hotel price reasonableness (Hypothesis 8).

## 3. Methodology

### 3.1. Measures

To evaluate the research constructs, measures were adopted from existing literature [4,6,16,21,22,36,48,49,50]. Multiple measurement items for all of the study variables were utilized. Specifically, three items (i.e., availability, accessibility, and variety) were used to measure green spaces (e.g., “This hotel has a variety of green spaces like gardens, natural surroundings (river/lake/mountain/ocean), green rest areas, and a green lobby, inside and outside”). Based on previous studies, measurement was taken in three different areas, including the degree of ease for hotels to use green space, the degree of physical access to green space, and the variety of green environments. These items were rated on a seven-point Likert-type scale from “strongly disagree” (1) to “strongly agree” (7). To evaluate psychological resilience, three items were utilized (e.g., “Staying at this hotel helps me feel refreshed,” rated from “strongly disagree” [1] to “strongly agree” [7]). Attitude was assessed with four items (e.g., “For me, staying at this hotel is…” rated from “bad” [1] to “good” [7]). 

Brand-self connection was evaluated with two items (e.g., “To what extent do you feel that you are personally connected to this hotel?” rated from “not at all” [1] to “completely” [7]). To measure brand prominence, two items were utilized (e.g., “To what extent are your thoughts and feelings toward this hotel often automatic, coming to mind seemingly on their own?” rated from “not at all” [1] to “completely” [7]). Price reasonableness was assessed with three items (e.g., “For me, the price paid at this hotel is…” rated from “unreasonable” [1] to “reasonable” [7]). Lastly, to evaluate traveler retention, four items were used (e.g., “This hotel will be my first choice the next time I travel to this location,” rated from “strongly disagree” [1] to “strongly agree” [7]). All of these measures were incorporated into the survey questionnaire contained along with a description of the study. The questionnaire was pre-tested by hospitality academics and hotel practitioners and minor corrections were made based on the participants’ feedback. The survey questionnaire was further examined and finalized by three academic experts. 

### 3.2. Data Collection and Samples

A web-based survey method was used to collect data. The developed and tested questionnaire was sent to potential participants via e-mail. These potential participants were randomly chosen from the survey company’s database and were general hotel customers. To be eligible for survey participation, the respondents were required to have had at least one hotel-stay experience within the last 12 months. The survey participants were first requested to read the research description and the survey instructions carefully. They were then asked to provide the name of the hotel at which they had most recently stayed. Next, they were asked to complete the questionnaire based on their experiences at the hotel that they indicated. Using this approach, a total of 403 usable responses were collected and utilized for the analysis.

Of 403 respondents, 44.4% were male guests, and 55.6% female. The participants’ average room nights within the last 12 months were 11.56 days. The mean age was 35.67 years old. Concerning their annual income, 46.4% reported that their annual income is between 25,000–54,999$, followed by $55,000–$84,999 (28.3%), $85,000 or more (17.9%), and $24,999 or less (7.4%). Concerning the respondents’ education level, the majority of the participants indicated that they are 4-year college graduates (69.7%), followed by graduate-degree holders (18.9%), 2-year/some college graduates (8.2%), and high-school graduates or less (3.2%). Regarding the purpose of their hotel stay, most respondents reported that they were pleasure travelers (81.1%), followed by business travelers (18.4%), and other (0.5%).

## 4. Data Analysis and Results

### 4.1. Measurement Quality Assessment

The data were analyzed using the SPSS 22 and AMOS 22. A confirmatory factor analysis was initially conducted to evaluate the measurement quality. The results showed the goodness-of-fit statistics for the measurement model to be satisfactory (χ^2^ = 412.876, *df* = 147, χ^2^/*df* = 2.809, *p* < 0.001, RMSEA = 0.067, CFI = 0.952, IFI = 0.952, TLI = 0.938). The model’s composite reliability was also tested. The assessment revealed that the values (green spaces = 0.880; psychological resilience = 0.862; attitude = 0.875; brand-self connection = 0.867; brand prominence = 0.810; hotel price reasonableness = 0.856; traveler retention = 0.905) are all above the cut-off of 0.70 recommend by Hair et al. [51]. The internal consistency of the observed items for all constructs was therefore confirmed. Next, the average variance extracted values were calculated. The values (green spaces = 0.709; psychological resilience = 0.677; attitude = 0.637; brand-self connection = 0.765; brand prominence = 0.682; hotel price reasonableness = 0.666; traveler retention = 0.761) were all above Hair et al.’s [51] minimum threshold of 0.50. Moreover, these values were greater than the between-construct correlations (squared). Convergent and discriminant validity were therefore also evident. The details of the measurement model’s evaluation results are presented in Table 1 and Table 2.

### 4.2. Structural Model Assessment

**Figure 2:**
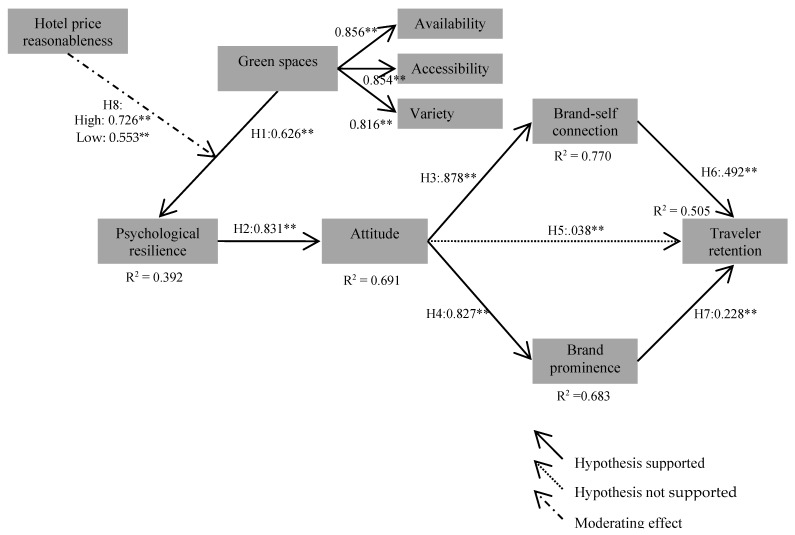
Results of the structural model and baseline model assessment (n = 403). Goodness-of-fit statistics for the structural model: χ^2^ = 381.640, *df* = 238, *p* < 0.001, χ^2^/*df* = 3.469, RMSEA = 0.078, CFI = 0.943, IFI = 0.943, TLI = 0.930. Goodness-of-fit statistics for the baseline model: χ^2^ = 593.956, *df* = 231, *p* < 0.001, χ^2^/*df* = 2.571, RMSEA = 0.063, CFI = 0.923, IFI = 0.924, TLI = 0.909, ** *p* < 0.01.

Next, a structural equation modeling was performed. The generated model showed adequate goodness-of-fit statistics (χ^2^ = 381.640, *df* = 238, *p* < 0.001, χ^2^/*df* = 3.469, RMSEA = 0.078, CFI = 0.943, IFI = 0.943, TLI = 0.930). The model generally had a satisfactory level of prediction power for retention, as it accounted for approximately 50.5% of the total variance in traveler retention. The model also explained approximately 77.0% and 68.3% of the variances in brand-self connection and brand prominence respectively. Additionally, approximately 69.1% of the variance in attitude was accounted for by its predictors. Moreover, green spaces explained about 39.2% of the total variance in psychological resilience. Figure 2 and Table 3 show the details of the structural model’s assessment results.

The hypothesized relationships were also tested. Our results showed that green spaces exert a significant influence on psychological resilience (β = 0.626, *p* < 0.01) and that attitude is a significant function of psychological resilience (β = 0.831, *p* < 0.01). This result supports Hypotheses 1 and 2. The impact of attitude was assessed next. Our findings revealed that attitude has a significant influence on brand-self connection (β = 0.878, *p* < 0.01) as well as brand prominence (β = 0.827, *p* < 0.01). However, attitude is not significantly associated with traveler retention (β = 0.038, *p* > 0.05). Hence, while Hypotheses 3 and 4 are supported, Hypothesis 5 is not supported. Our evaluation of the effect of brand-self connection and brand prominence showed that both brand-self connection (β = 0.492, *p* < 0.01) and brand prominence (β = 0.228, *p* < 0.05) has a significant influence on traveler retention. This result supports Hypotheses 6 and 7.

Next, the indirect impact of the study variables was examined. As shown in Table 4, green spaces have a significant indirect effect on attitude (β = 0.521, *p* < 0.01), brand-self connection (β = 0.457, *p* < 0.01), brand prominence (β = 0.430, *p* < 0.01), and traveler retention (β = 0.343, *p* < 0.01). Psychological resilience also has a significant indirect effect on brand-self connection (β = 0.730, *p* < 0.01), brand prominence (β = 0.687, *p* < 0.01), and traveler retention (β = 0.547, *p* < 0.01). Furthermore, attitude has a significant indirect effect on traveler retention (β = 0.620, *p* < 0.01). This result implies that psychological resilience, attitude, brand-self connection, and brand prominence play a mediating role in the proposed research model. Subsequently, the total effect of the research constructs was assessed. Our results showed that attitude has the greatest influence on traveler retention (β = 0.658, *p* < 0.01), followed by psychological resilience (β = 0.547, *p* < 0.01), brand-self connection (β = 0.492, *p* < 0.01), green spaces (β = 0.343, *p* < 0.01), and brand prominence (β = 0.228, *p* < 0.05). The details are presented in Table 4. 

### 4.3. Baseline Model Assessment and Test for Metric Invariance

To assess the effect of hotel price reasonableness, a grouping was performed. Based on the K-means analysis results, the responses concerning hotel price reasonableness were divided into two groups: high and low. The high price reasonableness group included 166 cases and the low price reasonableness group 237 cases. A baseline model where all loadings are equally restricted across high and low price reasonableness groups was generated. As can be seen in Table 5 and Figure 2, the model had acceptable goodness-of-fit statistics (χ^2^ = 593.956, *df* = 231, *p* < 0.001, χ^2^/*df* = 2.571, RMSEA = 0.063, CFI = 0.923, IFI = 0.924, TLI = 0.909). This model was then compared with the nested model in which the particular link (i.e., the relationship between green spaces and psychological resilience) was restricted to be equivalent between the two groups. The results of the chi-square test showed that the path from green spaces to psychological resilience differ significantly between the high and low groups of hotel price reasonableness (Δχ^2^ [1] = 4.754, *p* < 0.05). This result supports the proposed moderating effect of hotel price reasonableness. Accordingly, Hypothesis 8 is supported.

## 5. Discussion and Implications

Undoubtedly, offering healthy and natural physical surroundings to guests has become a vital issue in the hotel industry across the globe. This study explored the influence of hotels’ green spaces as nature-based solutions for psychological resilience, attitude, and brand attachment (brand-self connection and brand prominence) in the traveler retention process. The proposed theoretical framework in this study was wholly supported and it satisfactorily accounted for the total variance in traveler retention. The role of psychological resilience, attitude, and brand attachment as antecedents of retention and mediators were clearly identified. Furthermore, the role of hotel price reasonableness as a moderator—which fortifies the effect of green spaces on psychological resilience—was clearly illustrated. This study makes a critical contribution to existing knowledge concerning the variables that trigger guests’ decisions to return to a particular hotel and the internal relationships between the variables within the hypothesized conceptual framework. Moreover, this study offers an essential theoretical as well as practical contribution, as it provides a deeper understanding of the possible dissimilarity across high and low price reasonableness groups in the formation of traveler retention. In summary, all of the research objectives were attained successfully. 

Consistent with previous environmental behavior studies [3,22,52], this study emphasizes the significance of green spaces as essential constituents of nature-based solutions. Specifically, green spaces that are available both inside and outside of hotels were shown to be significant factors affecting guests’ psychological resilience in the traveler retention process. This implies that despite its criticality, relying solely on hotel services is not enough to fulfill guests’ needs or wants concerning their psychological resilience and mental health while staying at a hotel. Based on the results of this study, increasing green spaces for rest, leisure, and physical activities both inside and outside of hotels and increasing green items (e.g., trees, flowers, potted plants, or green decorations or decor) within the green spaces can be essential to meet guests’ needs to relieve mental anxiety and stress, improving refreshed feeling, and boosting psychological well-being. These factors are essential for traveler retention and eco-friendly hotel management. Therefore, the psychological wellbeing of guests and various positive effects achieved by securing green spaces inside and outside of the hotel can be cited as essential conditions for the sustainable management of hotels. Considering these results, hotel proprietors need to focus on maximizing the use of the hotel’s green spaces to improve guest service and thereby retention strategies. For example, boosting the availability, accessibility, and variety of green spaces in a hotel (e.g., gardens, natural surroundings like rivers, lakes, mountains, or oceans, green rest areas, and green lobbies) by investing various resources can be effective for eliciting positive responses and behaviors in guests.

Our findings also demonstrate that the total effect of attitude on traveler retention was significantly greater than that of the other research constructs. Attitude also exerted a considerable effect on brand-self connection and brand prominence. Being aware of the criticality of attitude in the traveler retention process, hotel practitioners need to make various efforts to improve guests’ positive attitude. According to Han and Yoon [29] and Verma et al. [30], encouraging customers to believe that consuming a certain product or service will produce positive outcomes for themselves is an effective means of increasing the level of customers’ favorable attitude toward the product or service. Therefore, informing guests about the diverse benefits of staying at a hotel where many green spaces/items/atmospherics are readily available (e.g., mental/physical health benefits) through various communication channels can be an efficient way to increase their positive attitude toward staying at the hotel, which maximizes brand-self connection, brand prominence, and retention. 

This study showed that the influence of psychological resilience on its subsequent factors is considerable. This result is of great theoretical importance, as it shows that psychological resilience is a crucial element when building a theoretical framework explaining the role of green spaces as nature-based solutions in traveler retention formation. To increase guests’ psychological resilience, hotel proprietors need to focus on creating a comfortable environment for their guests. Previous research indicated that customers’ mental health and psychological well-being can be improved when they form complementary relationships with relaxing/nature-friendly physical atmospheres [2,6]. This would reinforce the effect of green spaces on the retention process, ultimately leading to an enhanced retention rate. 

Results from the metric invariance verified that the link between green spaces and psychological resilience was significantly moderated by hotel price reasonableness. In particular, the strength of the relationship was greater for the high price reasonableness group (β = 0.726, *p* < 0.01) than for the low price reasonableness group (β = 0.553, *p* < 0.01). This implies that guests believe that a hotel’s green spaces and its performance help them to feel psychological resilience when their belief about price reasonableness at the hotel is strong. From a theoretical perspective, using price perception is therefore crucial in clearly identifying guests’ responses to a hotel’s green spaces. Existing conceptual models pertinent to guests’ behaviors can therefore be expanded by the integration of this cognitive concept with moderation characteristics. 

Practically, our findings imply that at similar levels of guest assessment regarding the performance of green spaces, they are more likely to feel refreshed and to relieve mental anxiety and stress in a high price reasonableness situation. Accordingly, hotel proprietors should actively address guests’ price perception to induce a maximum influence of the hotel’s green spaces on guests’ psychological resilience. Building on existing hotel literature, this study contributes crucial theoretical and managerial value, as our findings provides a deeper understanding of the behavioral discrepancy between two groups of price reasonableness in the hotel guests’ retention process.

Brand-self connection, brand prominence, attitude, and psychological resilience were shown to be important mediators in our hypothesized theoretical model. This means that the relationships among the study’s constructs are significantly influenced by these mediators are involved. This finding supports that brand-self connection and brand prominence is a bridge that mediates the attitude and retention relationship, that attitude is a bridge mediating the association between psychological resilience and brand attachment, and that psychological resilience is a bridge mediating the relationship between green spaces and attitude. Hospitality researchers should recognize the intricate and crucial mediating nature of the study variables. Based on this evidence, it is critical for researchers to utilize brand-self connection, brand prominence, attitude, and psychological resilience as mediators when developing a theory about guests’ responses to a hotel’s nature-based solutions and their purchase behaviors concerning the hotel’s product.

This study has a few limitations that may provide future study opportunities. First, our proposed theoretical framework was tested in the hotel context by using a data sample comprised of hotel guests. Accordingly, caution is required when generalizing our research findings to different tourism or consumer behavior sectors. Adopting a broader range of respondents would enhance the generalizability of future studies. Second, this research considered brand-self connection as a positive bond. Park et al. [16]) indicated that the negative brand-self dissociation is also a possible dimension of brand attachment. Future research should integrate such negative dissociation between the self and the brand into its proposed model. Third, hotel guests visit different hotels for different staying periods, different purposes (e.g., business purposes, attending meetings, leisure activities, and relaxing) in different regions by purchasing different package products. Therefore, future research needs to classify hotel guests in more specific categories and conduct verification with guests who choose hotels in various regions at various price ranges. Fourth, although this study focuses on the hospitality industry, measurement tools used in the urban environment, service industries, and cruise industry were utilized in this study. This is likely to result in errors that may occur from differences in the characteristics of the hospitality sector and other types of industries. Therefore, there is a need to use measurement tools unique to the hotel sector for future studies.

## 6. Conclusions

In conclusion, the theoretical basise of this study concerning the nature of “green spaces” and the associated human responses and behaviors in the hotel sector is fairly weak. This study helps us to better comprehend a hotel’s green spaces (availability, accessibility, and variety) and its possible influence on guests’ psychological, emotional, and conative responses and consumption behaviors. More specifically, the green space in hotels can positively increase the psychological resilience of customers, and such positive reactions of customers can be a great aid in forming attitudes toward the hotel and building positive branding. Such a clear understanding and perception of the importance of green spaces in hotels can be said to have presented a strategic direction for hotel operators to move forward, and a very concrete and meaningful strategy to incite positive customer attitudes toward the hotel. This study was the first in developing a sturdy framework of traveler retention by discovering the intricate associations among green spaces as nature-based solutions, psychological resilience, attitude, brand self-connection, brand prominence, and hotel price reasonableness. Despite its few limitations, this study offers high value and originality, advancing knowledge in hotel industry literature.

## Figures and Tables

**Table 1:** Measurement items and factor loadings (n = 403).

Items	Factor Loadings (Standardized)
Green spaces 1 Green spaces 2 Green spaces 3	0.856 0.856 0.814
Psychological resilience 1 Psychological resilience 2 Psychological resilience 3	0.884 0.803 0.777
Attitude 1 Attitude 2 Attitude 3 Attitude 4	0.812 0.822 0.785 0.772
Brand-self connection 1 Brand-self connection 2	0.851 0.898
Brand prominence 1 Brand prominence 2	0.777 0.872
Price reasonableness 1 Price reasonableness 2 Price reasonableness 3	0.716 0.903 0.818
Guest retention 1 Guest retention 2 Guest retention 3	0.869 0.924 0.821

**Table 2:** Measurement model assessment and correlations (n = 403).

Constructs	(a)	(b)	(c)	(d)	(e)	(f)	(g)	Mean (SD)	CR (AVE)
(a) Green spaces	1.000	–	–	–	–	–	–	4.883 (1.069)	0.880 (0.709)
(b) Psychological resilience	0.542 ^a^ (0.294) ^b^	1.000	–	–	–	–	–	4.953 (0.981)	0.862 (0.677)
(c) Attitude	0.468 (0.219)	0.601 (0.361)	1.000	–	–	–	–	5.507 (0.847)	0.875 (0.637)
(d) Brand-self connection	0.449 (0.210)	0.668 (0.446)	0.627 (0.393)	1.000	–	–	–	4.779 (1.057)	0.867 (0.765)
(e) Brand prominence	0.355 (0.126)	0.573 (0.328)	0.571 (0.326)	0.670 (0.449)	1.000	–	–	4.537 (1.088)	0.810 (0.682)
(f) Hotel price reasonableness	0.090 (0.008)	0.203 (0.041)	0.203 (0.041)	0.161 (0.026)	0.385 (0.148)	1.000	–	4.012 (1.045)	0.856 (0.666)
(g) Traveler retention	0.415 (0.172)	0.592 (0.350)	0.629 (0.396)	0.703 (0.494)	0.734 (0.539)	0.287 (0.082)	1.000	4.863 (1.036)	0.905 (0.761)

Goodness-of-fit statistics for the measurement model: χ^2^ = 412.876, *df* = 147, χ^2^/*df* = 2.809, *p* < 0.001, RMSEA = 0.067, CFI = 0.952, IFI = 0.952, TLI = 0.938, SD: standard deviation, CR: composite reliability, AVE: average variance extracted, ^a^ Correlations between variables are below the diagonal. ^b^ Squared correlations are within the parentheses.

**Table 3:** Structural model estimation (n = 403).

Proposed Paths				Coefficients	*t*-Values
H1	Green spaces	→	Psychological resilience	0.626	11.042 **
H2	Psychological resilience	→	Attitude	0.831	12.964 **
H3	Attitude	→	Brand-self connection	0.878	13.150 **
H4	Attitude	→	Brand prominence	0.827	13.083 **
H5	Attitude	→	Traveler retention	0.038	0.243
H6	Brand-self connection	→	Traveler retention	0.492	3.878 **
H7	Brand prominence	→	Traveler retention	0.228	2.222 *
Total variance explained: R^2^ for Traveler retention = 0.505 R^2^ for brand-self connection = 0.770 R^2^ for brand prominence = 0.683 R^2^ for attitude = 0.691 R^2^ for psychological resilience = 0.392					

Goodness-of-fit statistics for the structural model: χ^2^ = 381.640, *df* = 238, *p* < 0.001, χ^2^/*df* = 3.469, RMSEA = 0.078, CFI = 0.943, IFI = 0.943, TLI = 0.930. * *p* < 0.05, ** *p* < 0.01.

**Table 4:** Indirect impact and total impact assessment (n = 403).

Indirect Effects of	on				
	Psychological Resilience	Attitude	Brand-Self Connection	Brand Prominence	Traveler Retention
Green spaces	–	0.521 **	0.457 **	0.430 **	0.343 **
Psychological resilience	–	–	0.730 **	0.687 **	0.547 **
Attitude	–	–	–	–	0.620 **
Total impact on traveler retention: β green spaces = 0.343 ** β psychological resilience = 0.547 ** β attitude = 0.658 ** β brand-self connection = 0.492 ** β brand prominence = 0.228 *					

Goodness-of-fit statistics for the structural model: χ^2^ = 381.640, *df* = 238, *p* < 0.001, χ^2^/*df* = 3.469, RMSEA = 0.078, CFI = 0.943, IFI = 0.943, TLI = 0.930, * *p* < 0.05, ** *p* < 0.01.

**Table 5:** Baseline model estimation and invariance test results.

Paths	High Group of Hotel Price Reasonableness (n = 166)		Low Group of Hotel Price Reasonableness (n = 237)		Baseline Model (Freely Estimated)	Nested Model (Constrained to be Equal)
	β	t-Value	β	t-Value		
Green spaces → Psychological resilience	0.726	9.694 **	0.553	7.596 **	χ^2^ (231) = 593.956	χ^2^ (232) = 598.710

Chi-square difference test: a Δχ2 (1) = 4.754, *p* < 0.05 (H8: Supported), Goodness-of-fit statistics for the baseline model: χ^2^ = 593.956, df = 231, *p* < 0.001, χ2/df = 2.571, RMSEA = 0.063, CFI = 0.923, IFI = 0.924, TLI = 0.909, ** *p* < 0.01.
